# Effects of emergency/nonemergency cervical cerclage on the vaginal microbiome of pregnant women with cervical incompetence

**DOI:** 10.3389/fcimb.2023.1072960

**Published:** 2023-03-09

**Authors:** Yunshan Xiao, Shiting Huang, Weiwei Yu, Yan Ni, Danni Lu, Quanfeng Wu, Qin Leng, Ting Yang, Meilan Ni, Jingxian Xie, Xueqin Zhang

**Affiliations:** ^1^ Department of Obstetrics, Women and Children’s Hospital, School of Medicine, Xiamen University, Xiamen, China; ^2^ Xiamen Key Laboratory of Basic and Clinical Research on Major Obstetrical Diseases, Xiamen, China; ^3^ Xiamen Clinical Research Center for Perinatal Medicine, Xiamen, China

**Keywords:** cervical cerclage, cervical incompetence, vaginal microbiota, PTB, preterm birth

## Abstract

**Background:**

Evaluation of the therapeutic effects of cerclage on preterm birth (PTB) caused by cervical incompetence remains challenging. The vaginal microbiome is associated with preterm births. Thus, this study aimed to analyse the vaginal microbiota of patients with cervical incompetence, explore the relationship between the composition of the vaginal microbiota before cervical cerclage and at term delivery, and assess the effect of cervical cerclage on the vaginal microbiota.

**Methods:**

Patients (n = 30) underwent cerclage performed by the same surgical team. Vaginal swabs were obtained pre-surgery and seven days post-surgery. A gestational age-matched cohort of healthy pregnant women (n = 20) (no particular abnormality during pregnancy, delivery at term) was used as the control group and sampled during a comparable pregnancy. All collected vaginal swabs were analysed by 16S rRNA gene sequencing.

**Results:**

When comparing the healthy control and cervical cerclage groups, the enriched microorganism in the healthy controls was *G. Scardovia*, and the enriched microorganism of the cerclage was *G. Streptococcus*. α diversity was significantly increased in patients who received cerclage with preterm delivery compared with those with full-term delivery, and the enriched microorganism was *F. Enterococcus*. A comparison before and after nonemergency cerclage suggested that the enriched microorganisms were *G. Lactobacillus* and *F. Lactobacillaceae* before surgery. After nonemergency cerclage, the enriched microorganisms were *F. Enterobacteriaceae* and *C. Gammaproteobacteria*. Vaginal microbiota diversity significantly increased, and the proportion of women with *Lactobacillus* spp.-depleted microbiomes increased after emergency cerclage. Significant differences in β diversity were found between the groups. Before the emergency cerclage, the enriched microorganisms were *G. Lactobacillus*, *O. Alteromonadales*, and *P. Firmicutes*. After emergency cerclage, the enriched microorganisms were *P. Actinobacteria*, *C. Actinobacteria*, *P. Proteobacteria*, *F. Bifidobacteriaceae*, *O. Bifidobacteriales*, *G. Gardnerella*, and *G. Veillonella*.

**Conclusion:**

Cerclage (particularly emergency cerclage) may alter the vaginal microbiota by increasing microbiota diversity, decreasing vaginal *Lactobacillus* abundance, and increasing the abundance of pathogenic bacteria that are not conducive to pregnancy maintenance, thereby affecting surgical efficacy. Therefore, the role of the vaginal microbiome should be considered when developing treatment strategies for pregnant women with cervical incompetence.

**Clinical trial registration:**

https://www.chictr.org.cn, identifier ChiCTR2100046305.

## Introduction

Preterm birth is a serious medical concern worldwide. Globally, approximately 15 million premature infants are born annually and 1 million preterm birth-related infant deaths occur (Liu et al., 2016). Preterm birth is a major cause of perinatal death and the birth of disabled children, seriously influencing family happiness and social harmony. Therefore, comprehensive prevention and intervention strategies must be explored. Spontaneous preterm birth (sPTB) accounts for about 40–45% of preterm births (Goldenberg et al., 2008). Cervical incompetence (CIC) is the main risk factor for sPTB. The clinical diagnosis of CIC was based on the history of sPTB, clinical manifestations (no significant uterine contractions, progressive cervical shortening, or cervical canal dilation), or ultrasound findings. This mechanism may be associated with a structural defect or dysfunction of the sphincter in the cervical isthmus, resulting in the failure to maintain pregnancy to term. The prematurity rate of CIC patients is 3.3 times higher than that of non-CIC patients, accounting for 40–50% of sPTB cases (Slattery and Morrison, 2002). Thus, clinical intervention for CIC is essential to prevent preterm labour. According to the preterm birth prevention guidelines published by the American College of Obstetricians and Gynecologists (ACOG), cerclage is the only effective surgical option for CIC (ACOG, 2014).

The proportion of preterm births due to reproductive tract infections is approximately 25–40% of the total number of preterm births (Tsonis et al., 2020). As an invasive surgery, cerclage may increase the risk of infection and dysbiosis of the vaginal microbiota. Some patients delivered at full term, whereas others delivered shortly after surgery. This phenomenon is also associated with subclinical infections (Kanninen et al., 2011; Bauer et al., 2020; Bayar et al., 2020).

How do we identify ‘hidden’ and key infectious factors? It is essential to determine “who benefits from cervical cerclage.” Traditional pathogen detection methods have limited capabilities for identifying reproductive tract infections and vaginal microbiomes. Using 16S rRNA gene sequencing, high-throughput detection of vaginal microbial-exclusive nucleic acid fragments at the genus level, and detailed analysis of vaginal microbiota can provide a potent analytical tool for understanding the pathogenesis of preterm birth and predicting its risk of preterm birth (Ansari et al., 2021; Dunlop et al., 2021; Flaviani et al., 2021; Kumar et al., 2021; Yan et al., 2022). It is reported that a machine learning approach based on vaginal microbiota high-throughput sequencing analysis, combined with cervical canal length and white blood cell count, was used to establish a prediction model for preterm birth that exhibited a high predictive capability, with an area under the curve (AUC) of 0.84 (Park et al., 2022).

Therefore, the influence of the vaginal microbiome should be considered when assessing the efficacy of cerclage in preterm births. Moreover, it is essential to clarify the cervical support function of cerclage to reduce the risk of vaginal microbiota dysbiosis.

## Materials and methods

### Participants and sampling procedures

**Figure 1:**
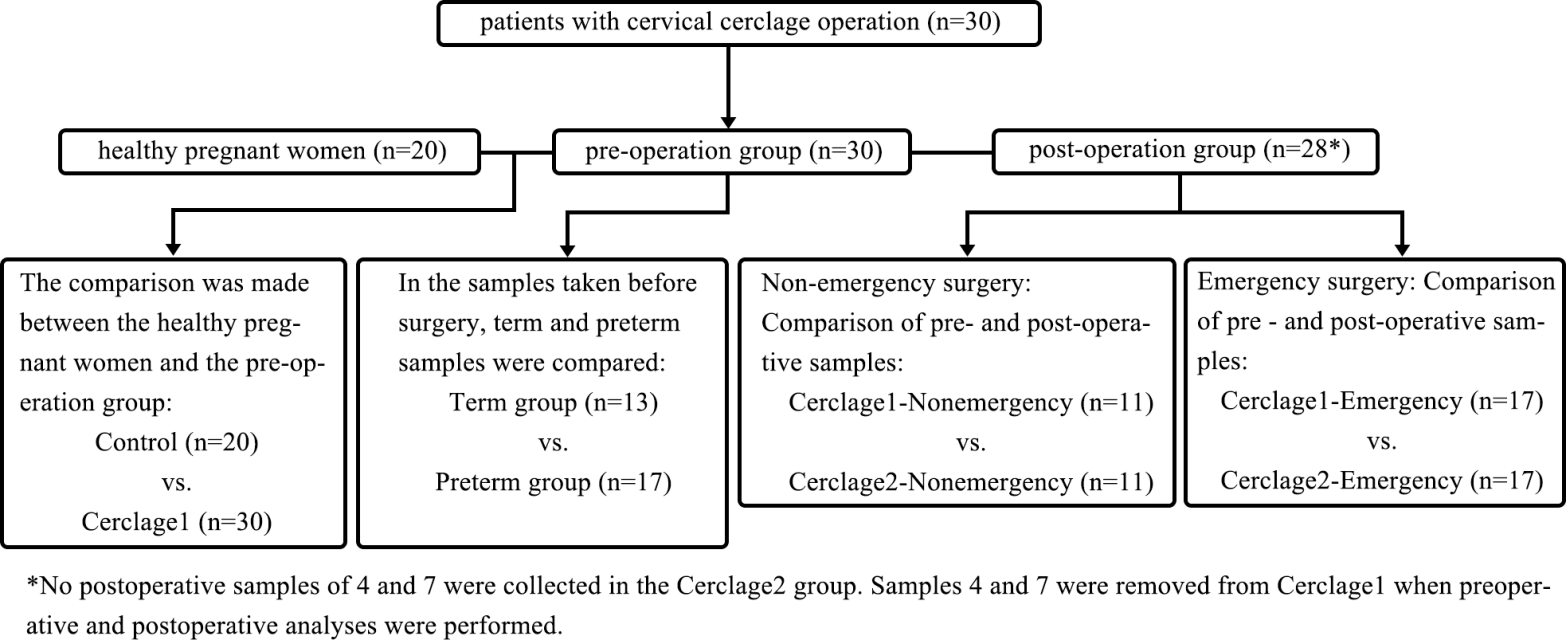
The workflow of study design.

This study used a case-control method and was approved by the Ethics Committee of the Xiamen Maternity and Child Health Care Hospital (XMCH) (Approval No. KY-2020-007). Written informed consent was obtained from all the participants. The study design and sampling plan are shown in **Figure 1**, **Table 1**, and **Supplementary Table 1**. The study was registered in the Chinese Clinical Trial Registration Database (https://www.chictr.org.cn; Registration No. ChiCTR2100046305). The participants’ data were collected from the hospital’s electronic record system. The inclusion criteria were as follows: women undergoing regular prenatal examination and delivery in XMCH from January 2020 to September 2021; women aged 20–40 years old; no use of antibiotics, progesterone, or immunosuppressants within one month before initial sampling; no sexual intercourse within 48 h; and no vaginal irrigation or medication. The exclusion criteria were as follows: severe disease; foetal chromosomal abnormalities or congenital malformations; occurrence of fever or diarrhoea within one week before sampling; and chorioamnionitis or other maternal infections, such as urinary infections and perianal abscesses.

**Table 1:** Pregnant women’s baseline demographic and clinical data undergoing cerclage.

Case No	Age	Gravida	Para	Cerclage indication	Cerclage weeks	Delivery weeks	Perinatal survival
1	32	1	0	nonemergency	15+1	40+2	yes
2	29	2	0	nonemergency	24+3	38+1	yes
3	39	4	1	nonemergency	24+1	39+2	yes
4	32	2	0	nonemergency	17+6	32+3	yes
5	34	5	1	nonemergency	24+1	34+2	yes
6	31	5	1	nonemergency	14+5	28+3	yes
7	39	2	0	nonemergency	25+2	32+3	no
8	34	1	0	nonemergency	22+4	25+3	no
9	31	3	0	nonemergency	23+3	30	yes
10	31	3	0	nonemergency	24+6	37+2	yes
11	26	6	0	nonemergency	25+3	38+1	yes
12	26	5	0	emergency	21+2	39+3	yes
13	28	1	0	emergency	24+2	36+2	yes
14	31	1	0	emergency	26+3	40+1	yes
15	34	6	1	emergency	23+3	24+2	no
16	33	2	0	emergency	26	38+6	yes
17	29	2	0	emergency	26	39+6	yes
18	28	1	0	emergency	23+6	38+5	yes
19	34	2	0	emergency	25	30+4	yes
20	25	2	0	emergency	24+3	40+1	yes
21	25	3	0	emergency	24	24+4	no
22	33	2	1	emergency	24	39+4	yes
23	35	1	0	emergency	22+5	24+2	no
24	32	1	0	emergency	24+4	26	no
25	29	3	1	emergency	28+4	38+1	yes
26	32	1	0	emergency	24+5	33+5	yes
27	27	2	0	emergency	18+4	31+3	yes
28	30	1	0	emergency	22+3	36+3	yes
29	33	1	0	emergency	24+5	29+3	yes
30	32	1	0	emergency	25+1	26+2	no

Indications for cerclage can be summarised based on one of the following conditions: i) nonemergency cerclage, including a history of medical indication (one or more painless dilatations of the cervix without uterine contraction and then abortion or premature delivery in second/third trimesters) and ultrasound indication (the cervical canal length is less than 25 mm during the second trimester); ii) emergency cerclage, including cervical canal dilation, no noticeable contractions, and with or without amniotic membrane sac exposure to the outer cervix. The patients underwent McDonald’s cerclage with braided Mersilene 5-mm tape. The Trendelenburg position was used to relieve pressure on the membranes of all patients under spinal anaesthesia. The membranes were gently pushed back into the internal os with a sponge stick or Foley balloon after gently pulling the cervical lips with ring forceps. Sutures were placed near the vaginal-cervical junction. Once the suture closed the internal os or endocervical canal, alternating knots were tied to prevent slippage.

Patients with indications for cerclage (n = 30) underwent cerclage performed by the same surgical team. Vaginal swabs were obtained pre-surgery and seven days post-surgery. The sample before cerclage surgery was defined as Cerclage1. After cerclage surgery, the sample was defined as Cerclage2. A gestational age-matched cohort of healthy pregnant women (n = 20) (no particular abnormality during pregnancy, without CIC, delivery at term; **Table S1**) was used as the control group and sampled during comparable pregnancy (14–28 weeks). Vaginal sampling was performed by dilating the vagina with a dilator and then collecting a sample without touching anything other than the sampling site, preventing sample contamination. At each time point, vaginal swabs were sampled from the posterior fornix under direct visualisation using swabs used for 16S rRNA gene sequencing with the BD CultureSwab MaxV system (Becton, Dickinson Company, Franklin Lakes, NJ, USA). The swabs were stored at -80°C.

### Vaginal microecological detection and bioinformatic analysis

**Figure 2:**
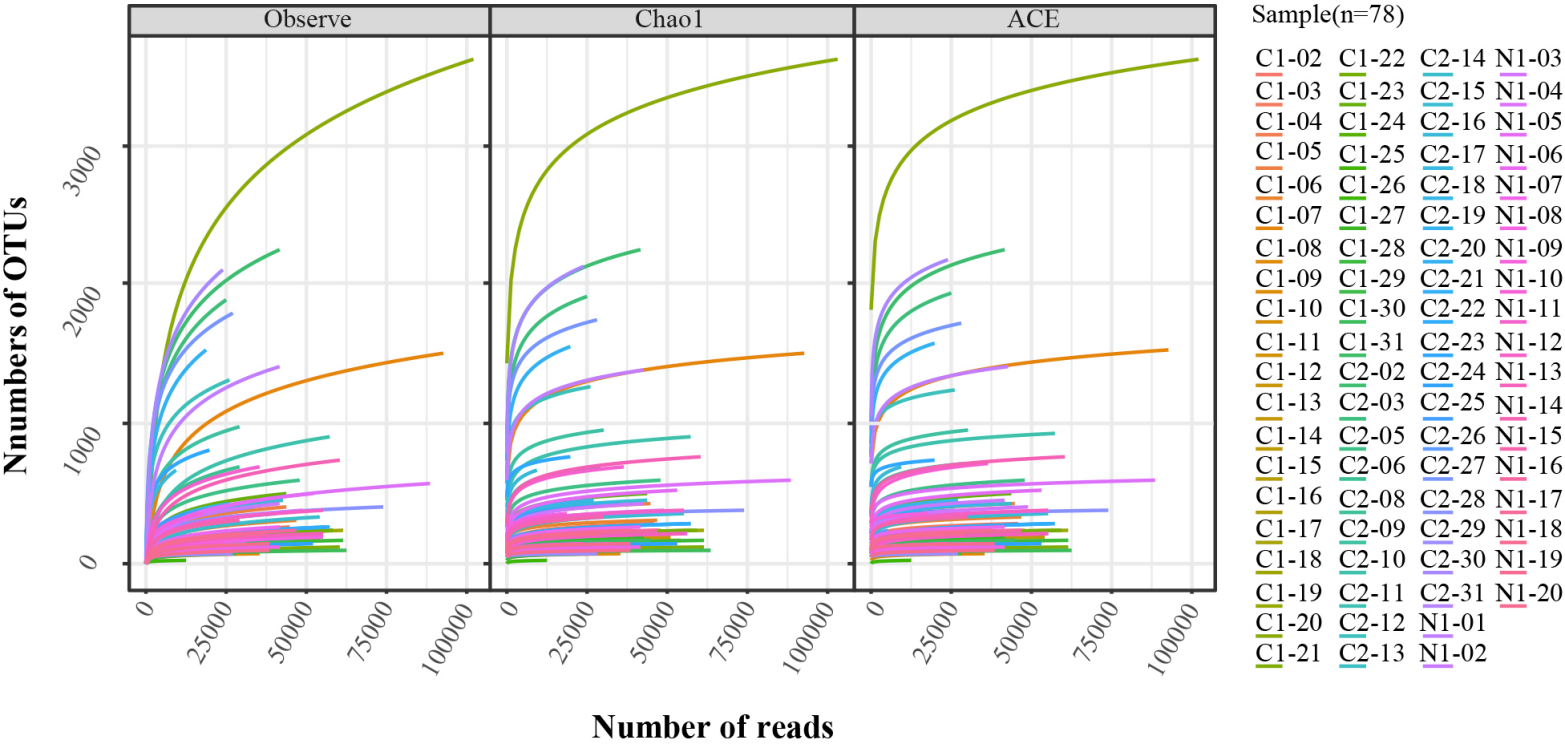
Rarefaction analysis of V3-V4 MiSeq sequencing reads of the 16S rRNA gene in all samples (n = 78). The Observe, Chao1, and ACE were used in the analysis.

Genomes were extracted from all the vaginal swab specimens using a BIOG DNA Swab Kit (BAIDAI, cat. #51029), according to the manufacturer’s instructions. The PCR of the 16SrRNA V3-V4 region was amplified by a 16S universal primer [forward primer: 5′-CCTAYGGGRBGCASCAG-3′ and reverse primer: 5′-GGACTACNNGGGTATCTAAT-3′]. PCR product purification, adapter ligation, DNA library purification, and quantitative quality control were performed. An Illumina NovaSeq (Illumina Inc., Chicago, IL, USA) was used as the sequencing platform. The effective data quantity was analysed using the rarefaction curve in **Figure 2**. Data quality control: Q30 > 80%.

Sequences were demultiplexed using Quantitative Insights into Microbial Ecology (QIIME1 version 1.8.0) and operational taxonomic units (OTUs) were generated using the UCLUST (version 1.8.0) denoising single plugin. Deduplication and standard quality filtering procedures were performed using default settings (**Table S2**, Bokulich et al., 2012). Cluster analysis of the high-quality tags revealed the required sequence consistency of the OTUs. The dominant sequences of OTUs were then compared using the Greengenes database version 13_8_99 (http://greengenes.microbio.me/greengenes_release/gg_13_8_otus/) to obtain species notes.

Bacteria were identified to the genus level using two variable regions of the gene 16s rRNA. A relative abundance bar plot was drawn based on the relative abundance of four *Lactobacillus* species and bacteria in which the abundance was more than 200 reads at the genus level. BLAST analysis yielded annotation results at the L. species level (Karaer et al., 2021). Three clusters were identified and characterised based on the abundance of *Lactobacillus* spp.: dominant (75–100%), intermediate (50–75%), and depleted (0–50%).

### Statistical analysis

The microbial diversity of individual samples was assessed by the alpha diversity index using the R package MicrobiotaProcess (v 1.2.2), and statistical analysis was performed using the Wilcoxon rank-sum test and Wilcoxon signed rank test. Beta diversity was determined as the distance between the microbial communities based on the Weighted UniFrac distance. The distances were visualised using principal coordinate analysis (PCoA). Differences based on beta diversity among the groups were calculated using the R package vegan (v 2.5-7) with Anosim, Mrpp, Adonis, and Amova. A two-sided *P* < 0.05 was considered statistically significant. Intergroup differential species were detected using linear discriminant analysis effect size (LEFSe), and a linear discriminant analysis (LDA) score > 4 was considered significant (Segata et al., 2011 Genome Biology, 12).

## Results

### Clinical characteristics of the study subjects and pregnancy outcome

The baseline data of pregnant women who underwent cerclage (including age, gravida/para ratio, cerclage indication, cerclage weeks, delivery weeks, and risk of perinatal death) are shown in **Table 1**. Among the 30 pregnant women who underwent cerclage, 13 delivered at term and 17 delivered preterm. There were 11 and 19 patients in the nonemergency and emergency cerclage groups, respectively. Among the nonviable foetuses, 6 had very early preterm births (delivery before 27 weeks) and one had excessive umbilical cord torsion with asphyxia. The clinical data of the gestational age-matched control group of pregnant women are shown in **Supplementary Table 1**.

### Characteristics of vaginal microbial communities before cervical cerclage compared with the control group

**Figure 3:**
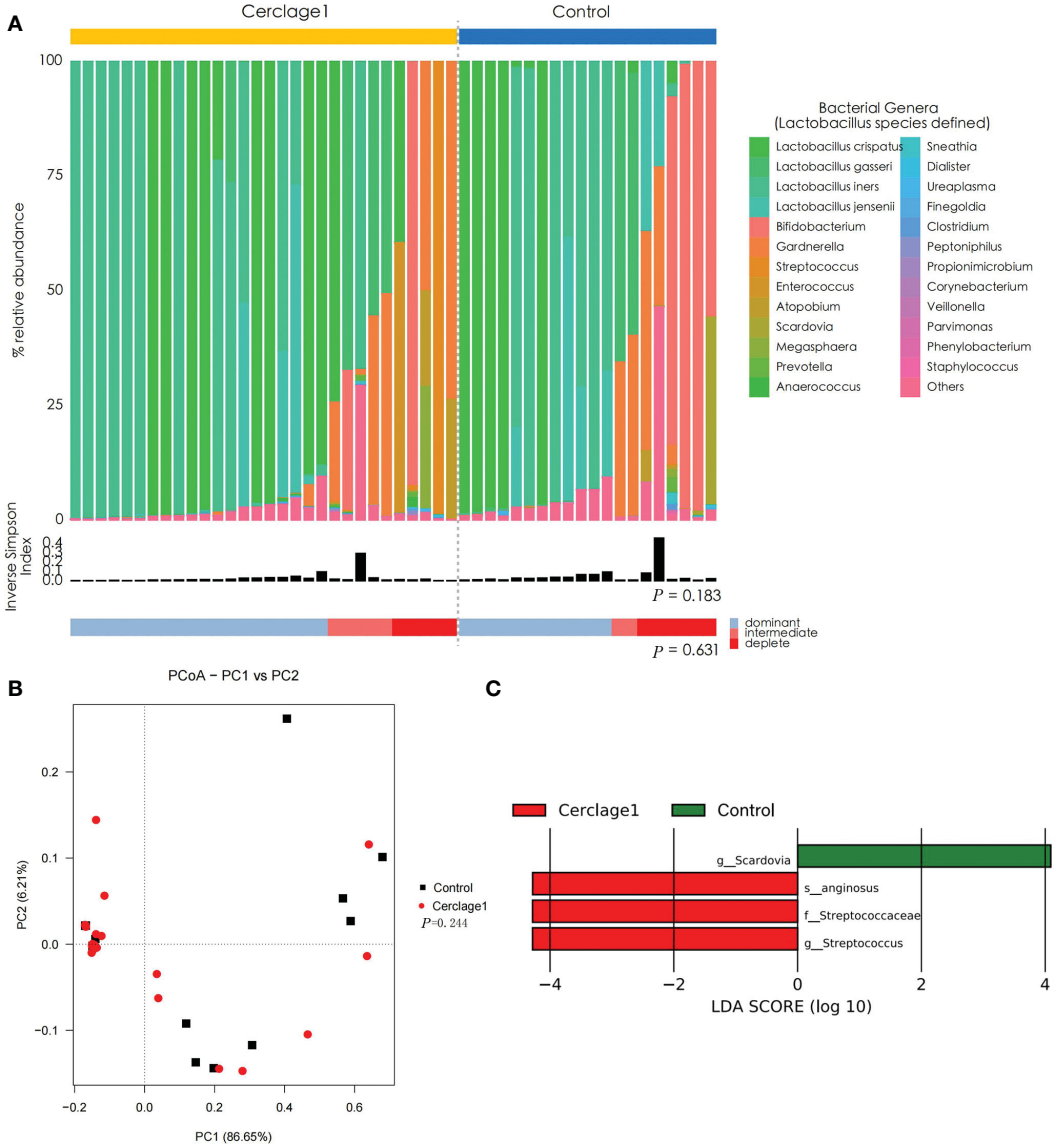
Characteristics of vaginal microbial communities before cervical cerclage compared with the control group. **(A)** The microbial community composition of the Cerclage1 (n = 30) and the control (n = 20) groups was analysed at the genus level, with the classification level for *Lactobacillus* spp. being at the species level. The black bars indicate the Inverse Simpson Index. The samples can be classified based on the relative abundance levels of *Lactobacillus* spp. as depleted (< 50%, red), intermediate (50% to 75%, pink), and dominant (>75%, blue). **(B)** PCoA analysis was performed on two groups, Cerclage1 (n = 30, red dots) and control (n = 20, black squares). The first and second principal coordinates are displayed on the horizontal and vertical axes respectively. The percentage value represents the contribution of each principal coordinate to the dissimilarity in the sample matrix data. **(C)** LEfSe analysis was performed to compare the Cerclage1 (n = 30, red) and control (n = 20, green) groups. Significant features were identified using an LDA score threshold of [(log10) > 4 or < -4] and a *p*-value cutoff of 0.05.

The proportion of women with *Lactobacillus* spp.-depleted microbiome was similar between the control and Cerclage1 groups [5/30 (16.7%) vs. 6/20 (40%), respectively; *P* = 0.631]. The α diversity of vaginal microbiota was also similar (inverse Simpson index: 0.290 vs. 0.241 for the control and Cerclage1 groups, respectively; *P* = 0.183) (**Figure 3A**). According to the results of PCoA, there was no significant difference in diversity between the two groups (Adonis analysis, *P* = 0.244) (**Figure 3B**). LEfSe analysis revealed that the enriched microorganism in the control group was *G. Scardovia.* The enriched microorganisms in the cerclage group were *F. Streptococcaceae* and *G. Streptococcus* (**Figure 3C**).

### Comparison of characteristics of vaginal microbial communities before cervical cerclage between the term and preterm groups

**Figure 4:**
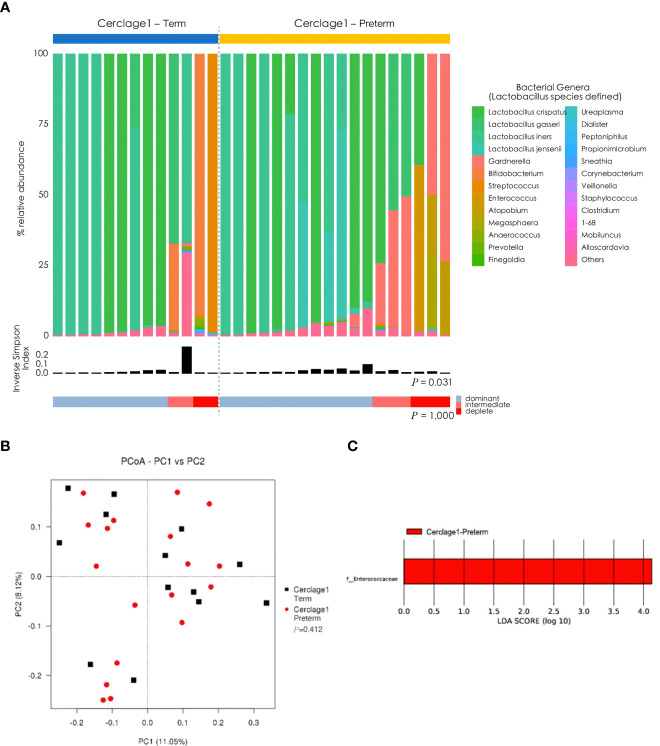
Comparison of characteristics of vaginal microbial communities before cervical cerclage between term and preterm groups. **(A)** The microbial community composition of the Cerclage1-Preterm (n = 17) and Cerclage1-Term (n = 13) groups was analysed at the genus level, with the classification level for *Lactobacillus* spp. being at the species level. The black bars indicate the Inverse Simpson Index. The samples can be classified based on the relative abundance levels of *Lactobacillus* spp. as depleted (< 50%, red), intermediate (50% to 75%, pink), and dominant (>75%, blue). **(B)** PCoA analysis was performed on two groups, Cerclage1-Preterm (n = 17, red dot) and Cerclage1-Term (n = 13, black square). The first and second principal coordinates are displayed on the horizontal and vertical axes respectively. The percentage value represents the contribution of each principal coordinate to the dissimilarity in the sample matrix data. **(C)** LEfSe analysis was performed to compare the Cerclage1-Preterm (n = 13, red) and Cerclage1-Term (n = 13, green) groups. Significant features were identified using an LDA score threshold of [(log10) > 4 or < -4] and a *p*-value cutoff of 0.05.

Compared with the Cerclage1-Term group, the α diversity of vaginal microbiota in the Cerclage1-Preterm group significantly increased (inverse Simpson index: 0.14 vs. 0.31, respectively; *P* = 0.031), and the proportion of *Lactobacillus* spp.-depleted microbiome was similar [2/13 (15%) vs. 3/17 (18%) for the Cerclage1-Term and Cerclage1-Preterm groups, respectively; *P* = 1.000] (**Figure 4A**). According to the PCoA, there was no significant difference in β diversity between the two groups (Adonis analysis, *P =* 0.412) (**Figure 4B**), and *F. Enterococcus* was relatively enriched in the vaginal microbiota in the Cerclage1-Term group (**Figure 4C**).

### Comparison of vaginal microbial communities before and after nonemergency cerclage

**Figure 5:**
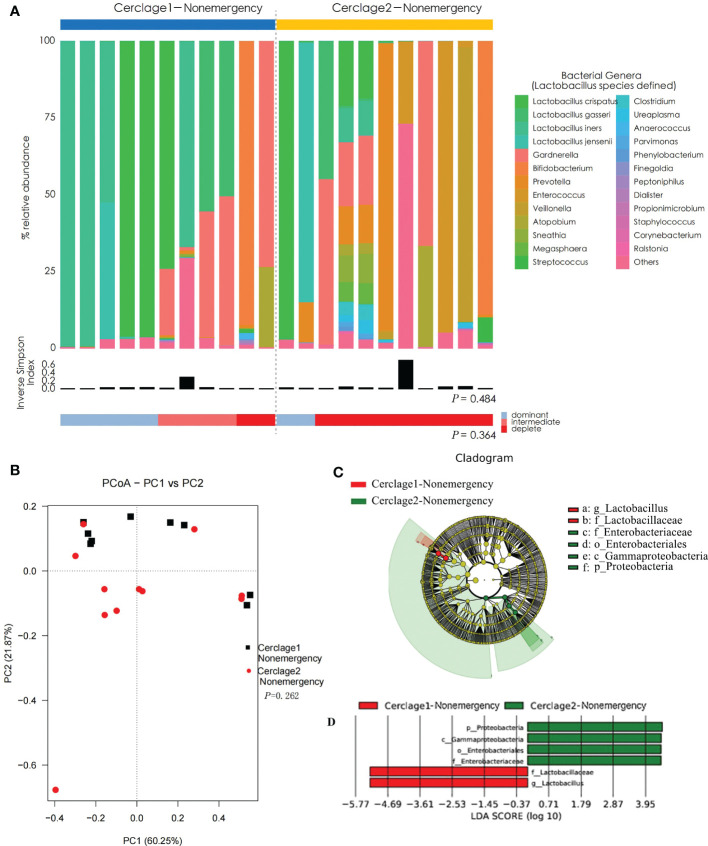
Comparison of vaginal microbial communities before and after nonemergency cerclage. **(A)** The microbial community composition of the Cerclage1-Nonemergency (n = 11) and Cerclage2-Nonemergency (n = 11) groups was analysed at the genus level, with the classification level for *Lactobacillus* spp. being at the species level. The black bars indicate the Inverse Simpson Index. The samples can be classified based on the relative abundance levels of *Lactobacillus* spp. as depleted (< 50%, red), intermediate (50% to 75%, pink), and dominant (>75%, blue). **(B)** PCoA analysis was performed on two groups, Cerclage1-Nonemergency (n = 11, black square) and Cerclage2-Nonemergency (n = 11, red dot). The first and second principal coordinates are displayed on the horizontal and vertical axes respectively. The percentage value represents the contribution of each principal coordinate to the dissimilarity in the sample matrix data. **(C)** LEfSe cladograms were generated for pairwise comparisons of the Cerclage1-Nonemergency (n=11, red) and Cerclage2-Nonemergency (n=11, green) groups. The cladograms illustrate the taxonomic hierarchy, with rings representing different levels of classification, ranging from phyla (innermost ring) to genera (outermost ring), and each circle represents a member within that level. The taxa at each level are shaded in either green (Cerclage2-Nonemergency) or red (Cerclage1-Nonemergency) to indicate their abundance (LDA score > 4 or < -4; *P* < 0.05). **(D)** LEfSe analysis was performed to compare the Cerclage1-Nonemergency (n = 11, red) and Cerclage2-Nonemergency (n = 11, green) groups. Significant features were identified using an LDA score threshold of [(log10) > 4 or < -4] and a *p*-value cutoff of 0.05.

There was no significant difference in α diversity of vaginal microbiota between Cerclage1-Nonemergency (n = 11) and Cerclage2-Nonemergency (n = 11) (inverse Simpson index: 0.29 vs. 0.40, respectively; *P* = 0.484). The proportion of women with *Lactobacillus* spp.-depleted microbiome was similar [2/11 (18%) vs. 3/11 (27%) for the Cerclage1-Nonemergency and Cerclage2-Nonemergency groups, respectively; *P* = 0.364] (**Figure 5A**). According to the results of PCoA, there was no significant difference in diversity between the two groups (Adonis analysis, *P* = 0.262) (**Figure 5B**). LEfSe analysis showed that the enriched microorganisms of Cerclage1-Nonemergency were *G. Lactobacillus* and *F. Lactobacillaceae*; and the enriched microorganisms of Cerclage2-Nonemergency were *P. Proteobacteria*, *O. Enterobacteriales*, *F. Enterobacteriaceae*, and *C. Gammaproteobacteria* (**Figures 5C, D**).

### Comparison of vaginal microbial communities before and after an emergency cerclage

**Figure 6:**
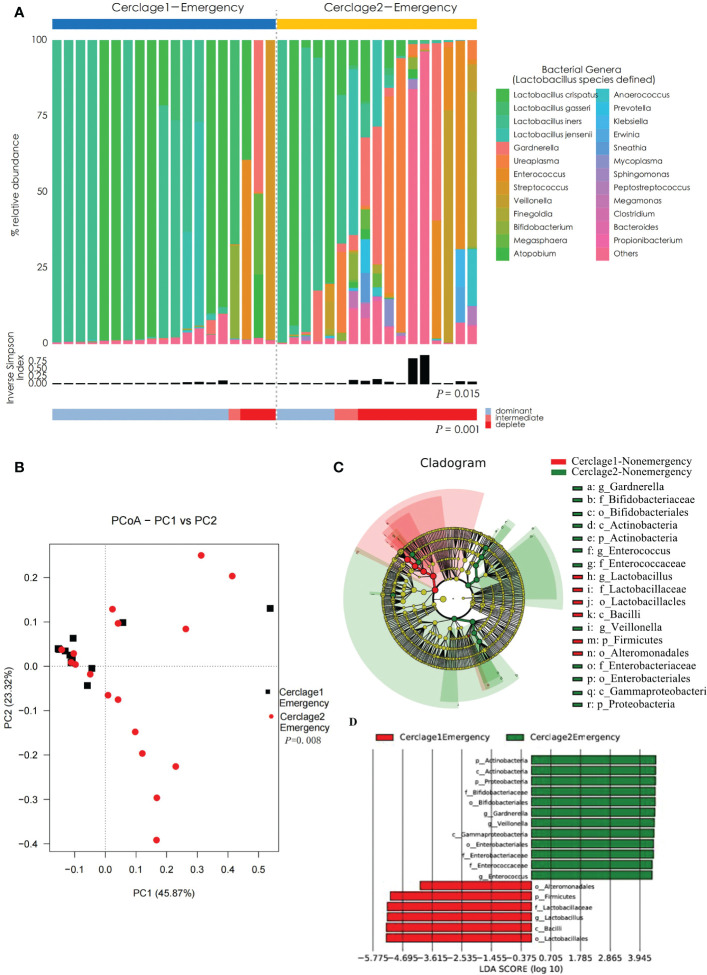
Comparison of vaginal microbial communities before and after an emergency cerclage. **(A)** The microbial community composition of the Cerclage1-Emergency (n = 17) and Cerclage2-Emergency (n = 17) groups was analysed at the genus level, with the classification level for *Lactobacillus* spp. being at the species level. The black bars indicate the Inverse Simpson Index. The samples can be classified based on the relative abundance levels of *Lactobacillus* spp. as depleted (< 50%, red), intermediate (50% to 75%, pink), and dominant (>75%, blue). **(B)** PCoA analysis was performed on two groups, Cerclage1-Emergency (n = 17, black square) and Cerclage2-Emergency (n = 17, red dot). The first and second principal coordinates are displayed on the horizontal and vertical axes respectively. The percentage value represents the contribution of each principal coordinate to the dissimilarity in the sample matrix data. **(C)** LEfSe cladograms were generated for pairwise comparisons of the Cerclage1-Emergency (n = 17, red) and Cerclage2-Emergency (n = 17, green) groups. The cladograms illustrate the taxonomic hierarchy, with rings representing different levels of classification, ranging from phyla (innermost ring) to genera (outermost ring), and each circle represents a member within that level. The taxa at each level are shaded in either green (Cerclage2-Nonemergency) or red (Cerclage1-Nonemergency) to indicate their abundance (LDA score > 4 or < -4; *P* < 0.05). **D)** LEfSe analysis was performed to compare the Cerclage1-Emergency (n = 17, red) and Cerclage2-Emergency (n = 17, green) groups. Significant features were identified using an LDA score threshold of [(log10) > 4 or < -4] and a *p*-value cutoff of 0.05.

Compared with Cerclage1-Emergency, the vaginal microbiota diversity significantly increased after emergency cerclage (inverse Simpson index: 0.21 vs. 0.43 for the Cerclage1-Emergency and Cerclage2-Emergency groups, respectively; *P* = 0.015). Additionally, the proportion of women with *Lactobacillus* spp.-depleted microbiome significantly increased [3/19 (16%) vs. 10/17 (59%) for the Cerclage1-Emergency and Cerclage2-Emergency groups, respectively; *P* = 0.001] (**Figure 6A**). According to the PCoA, the β diversity significantly varied between the two groups (Adonis analysis, *P* = 0.008) (**Figure 6B**). LEfSe analysis showed that the enriched microorganisms of Cerclage1-Emergency were *O. Alteromonadales*, *P. Firmicutes*, *F. Lactobacillaceae*, *G. Lactobacillus*, *O. Lactobacillales*, and *C. Bacilli*; and the Cerclage2-Emergency enriched microorganisms were *P. Actinobacteria*, *C. Actinobacteria*, *P. Proteobacteria*, *F. Bifidobacteriaceae*, *O. Bifidobacteriales*, *G. Gardnerella*, *G. Veillonella*, *C. Gammaproteobacteria*, *O. Enterobacteriales*, *F. Enterobacteriaceae*, *Enterococcaceae*, and *G. Enterococcus* (**Figures 6C, D**).

## Discussion

The vagina has a multilevel defense system composed of vaginal anatomy, microbiota, the body’s endocrine system, and local immune regulation. The vaginal microbiota is a critical component of the vaginal defense system. The maintenance of a normal pregnancy is closely associated with the balance of the vaginal microbiota. The vaginal microbiota of women with full-term delivery predominantly gravitates towards the *Lactobacillus*-dominated (LDOM) vaginal microbiota in the second and third trimesters. The risk of preterm birth increases when the microbiota is disturbed and manifests as a *Lactobacillus*-depleted microbiome (Petricevic et al., 2014; Short et al., 2021; Chan et al., 2022). *Lactobacillus* has a protective effect on pregnancy by inhibiting the activity of matrix metalloproteinases (MMPs), maintaining vaginal epithelial integrity, producing lactic acid to maintain a local low pH value, producing antimicrobial peptides to inhibit or kill harmful bacteria and viruses, affecting cytokine secretion, and regulating inflammatory reactions (Boris and Barbés, 2000; Calonghi et al., 2017; Parolin et al., 2018; Oliver et al., 2020). In the highly diverse vaginal microenvironment, various harmful microorganisms induce an immunoinflammatory response at the maternal–foetal interface and promote the occurrence of preterm birth (Wylie et al., 2018; iHMP, 2019). As previously reported, an abundance of *Gardnerella, Clostridiales, Bacteroidales, and Actinomycetales* leads to vaginal dysbiosis associated with preterm birth (Aagaard et al., 2012; Ansari et al., 2021). Other species that increase the risk of preterm birth include bacterial vaginosis-associated *Leptotrichia/Sneathia, Mobiluncus* spp.*, Mycoplasma* spp., and periodontal pathogens (e.g. *Porphyromonas gingivalis*) (Nelson et al., 2014; Wen et al., 2014; Lin et al., 2007).

The association between cervical incompetence and the vaginal microbiome has increasingly attracted the attention of researchers. Therefore, it is necessary to understand the characteristics of the vaginal microbiome in pregnant women treated with cerclage. A short cervix is associated with decreased abundance of vaginal *Lactobacillus* and increasingly strict and facultative anaerobes (Gerson et al., 2020). The abundance of vaginal *Lactobacillus* and its metabolite d-lactic acid could be predictors of a short cervix (Witkin et al., 2019). A reduced relative abundance of *Lactobacillus* has previously been associated with premature cervical dilation (Brown et al., 2019).

The present study did not find differences in the relative abundance of vaginal *Lactobacillus*, and α and β diversities in patients undergoing cerclage and healthy pregnant women during the same period. The significant contribution in the cerclage group is *G. Streptococcus* (**Figure 3C**). However, the functional role of *G. Scardovia* in the local vaginal environment has rarely been reported. It is one of the eight genera of the family *Bifidobacteriaceae*, possesses acid-producing activity, maintains an acidic environment of vaginal pH, and may be a potentially beneficial factor for pregnancy maintenance (Lugli et al., 2017). An association between *Streptococcus* and CIC has been reported. To that end, some researchers have demonstrated that screening and treatment of group B *Streptococcus* are effective in preventing CIC (Natale et al., 2013).

Cerclage is essential for maintaining pregnancy because it maintains cervical length and mucus plugs. It also provides a certain degree of mechanical bearing support for weakened cervical structures. The clinical effects of cervical cerclage are associated with the preoperative vaginal microbiome status. Some researchers have reported that excessive vaginal *Gardnerella* is correlated with unsuccessful cerclage and that high levels of vaginal *Lactobacillus* are associated with successful cerclage (Brown et al., 2019). They suggested that CIC patients should be divided into two groups: one group includes CIC patients with a weakened cervical structure and lack of capacity, indicating that they are highly appropriate for cerclage; the other group includes CIC patients with vaginal microbiota dysbiosis, and the cerclage effect is mainly unsatisfactory. Therefore, it is necessary to perform detailed vaginal microbiota testing to screen appropriate populations for cerclage. According to routine clinical practice, urine analysis and bacterial culture of vaginal secretions should be performed before cerclage. This indicates that urogenital infections should initially be treated with anti-infection medications. However, the above-mentioned traditional pathogen detection methods limit our understanding of infection and the vaginal microbiome. Using 16S rRNA gene sequencing, high-throughput detection of vaginal microbial-exclusive nucleic acid fragments at the gene level can be used to comprehensively explore vaginal microbiome characteristics, which is a valuable clinical evaluation tool.

The present study suggests that high preoperative vaginal microbiota diversity and high abundance of *Enterococcus* were factors influencing the failure of cerclage therapy. This indicates that cerclage is not suitable or should be considered after targeted intervention in this case (**Figure 4**). It is reported that *Enterococcus* may be a risk factor for catheter-related tract infection; the mechanism may be that invasive surgery destroys the local immune barrier of the host. *Enterococcus* adheres to cells, the extracellular matrix, and inert medical materials to form biofilms and secrete surface proteins, indicating its immune escape function and resistance to various cephalosporins and aminoglycoside antibiotics (O'Brien et al., 2016). The above-mentioned studies suggest that CIC patients with great vaginal microbiota diversity and a high abundance of *Enterococcus* are ineligible for cerclage.

It is well known that infection-related preterm birth is the leading cause of infant mortality and morbidity. Does this act as an invasive procedure that increases the risk of infection or vaginal microbiota dysbiosis? Some researchers believe that thick braided sutures induce dysbiosis of the vaginal microbiota (Kindinger et al., 2016; Pruski et al., 2021). The diversity of the vaginal microbiome increases after braided cerclage, with relatively rich Bacteroides and Clostridium and less *Lactobacillus*; it also affects the local vaginal metabolomic composition and increases the risk of preterm birth. Monofilament cerclages do not affect the potential bacterial composition of the vagina, thereby possessing more significant therapeutic efficacy than braided sutures (Brown et al., 2019). However, Battarbee et al. (2019) pointed out that the abovementioned study did not deeply analyse the potential effects of other clinical factors (e.g. maternal race, history of pregnancy, concurrent progesterone use, and cerclage criteria); thus, their conclusions were unreliable. Their clinical cohort study concluded that thick braided sutures (Mersilene 5-mm tape) outperformed, resulting in a longer delayed delivery time (longer duration of pregnancy), lower rates of chorioamnionitis, and neonatal intensive care unit admission. Braided sutures are commonly used at our institution. The reason is that the incidence of cervical cutting and suture fracture is higher after monofilament cerclage; however, the strength of braided sutures is immense, the incidence of cervical tear is low, and the material is easy to remove.

The present study revealed that the extent of influence of braided sutures on the vaginal microbiome was related to whether it was an emergency cerclage (**Figures 5**, **6**). There was no significant difference in vaginal microbiota on α and β diversities before and after surgery for women undergoing nonemergency cerclage. However, the postoperative abundance of *Lactobacillus* decreased, while the abundance of *Gammaproteobacteria, Proteobacteria*, and *Enterobacteriaceae* increased. Moreover, the diversity of vaginal microbiota significantly increased after emergency cerclage. The relative abundances of *Lactobacillus*, *Bacilli*, *Alteromonadales*, and *Firmicutes* were lower (while the relative abundances of *Actinobacteria, Proteobacteria, Bifidobacteriaceae, Gardnerella, Veillonella, Gammaproteobacteria, Enterobacteriaceae*, and *Enterococcus* were higher) after cerclage than before cerclage. It has been reported that *Gardnerella, Gammaproteobacteria*, and *Enterobacteriaceae* are closely associated with preterm birth (Conde-Agudelo & Romero, 2014; Doyle et al., 2014; van de Wijgert & Jespers, 2017; Gerson et al., 2022). *Veillonella* can promote lactate consumption and reduce local lactate levels, which are not conducive for vaginal microbiota homeostasis (Scheiman et al., 2019). As previously mentioned, a high abundance of *Enterococcu*s may increase the risk of preterm birth. Overall, the braided cervical cerclage may lead to dysbiosis of the vaginal microbiota. Compared with nonemergency cerclage, emergency cerclage had a more significant influence on the vaginal microbiota. However, whether this effect can be considered as an independent risk factor for treatment failure remains elusive, and further stratification and analysis with a larger sample size are required.

The aetiology of CIC remains elusive, and effective prevention methods need to be improved. Accurate evaluation of the effects of cerclage and screening of eligible cases for surgery are important clinical problems that should be urgently eliminated. To date, relevant studies have mainly concentrated on analysing clinical data collected from electronic medical records. In-depth analysis of the vaginal microbiome is required to clinically optimise interventional strategies.

This study has several limitations. For one, it required larger sample quantities and deviations. CIC’s clinical phenotypes and outcomes of CIC are heterogeneous (Golbasi et al., 2022). The analysis can be conducted more clearly by further stratification based on expanding the sample size. Additionally, this study was limited to the vaginal microbiota, lacked multifactor integration analysis, and did not consider the interaction between vaginal microorganisms and the host immune response (Fettweis et al., 2019; Al-Nasiry et al., 2020; Marangoni et al., 2021; Kwon and Lee, 2022).

## Conclusion

This study analysed the vaginal microbiomes of patients with cervical insufficiency, characterised the relationship between the composition of the vaginal microbiome before cerclage and that of healthy women, and assessed the effect of cerclage on the vaginal microbiome. We found that cerclage may influence the vaginal microbiota (especially emergency cerclage), resulting in an increase in microbiota diversity, decreased abundance of vaginal *Lactobacillus*, and increased abundance of pathogenic bacteria (which may be inconducive to pregnancy maintenance). This was a single-centre preliminary clinical study. Thus, larger sample sizes and multicentre collaborative studies are required. However, our findings provide valuable information in this field. Therefore, the role of vaginal microbiome should be considered when developing treatments for pregnant women with cervical incompetence.

## Data availability statement

The datasets presented in this study can be found at: https://www.ncbi.nlm.nih.gov/bioproject/PRJNA891026. The accession number is PRJNA891026. The raw data of the 16S rRNA sequence has been uploaded to NCBI (Accession to cite for these BioProject data: PRJNA891026).

## Ethics statement

The studies involving human participants were reviewed and approved by the ethics committee of the Xiamen Maternity and Child Health Care Hospital (XMCH). The patients/participants provided their written informed consent to participate in this study.

## Author contributions

YX is the first author who performed the study, collected data, and wrote/edited the manuscript. As the correspondence author, XZ performed this study’s design. SH analyzed the data. WY, YN, DL, QW, QL, TY, JX, and MN collected the samples and performed the experiments. All authors contributed to the article and approved the submitted version.
