# Supplementary material for: Effects of emergency/nonemergency cervical cerclage on the vaginal microbiome of pregnant women with cervical incompetence
Source: Front Cell Infect Microbiol. 2023 Mar 9;13:1072960. doi: 10.3389/fcimb.2023.1072960 (PMC10034410; doi:10.3389/fcimb.2023.1072960)
Supplement: Supplementary file 1 [file Table_1.docx]

**Supplementary table 1**

**1.1 pregnant women's clinical data of the gestational age-matched control group**

| Case NO | age | gravida | para | sampling week | delivery week | perinatal survival |
| --- | --- | --- | --- | --- | --- | --- |
| 1 | 31 | 2 | 1 | 15+1 | 37+3 | yes |
| 2 | 28 | 1 | 0 | 16+4 | 38+3 | yes |
| 3 | 29 | 1 | 0 | 18+4 | 39+6 | yes |
| 4 | 26 | 1 | 0 | 19+4 | 39 | yes |
| 5 | 28 | 1 | 0 | 22+3 | 39+6 | yes |
| 6 | 29 | 1 | 0 | 22+4 | 38+5 | yes |
| 7 | 34 | 3 | 1 | 23+3 | 39+1 | yes |
| 8 | 31 | 1 | 0 | 23+3 | 40+4 | yes |
| 9 | 31 | 3 | 1 | 24+1 | 40 | yes |
| 10 | 31 | 1 | 0 | 24+2 | 38+6 | yes |
| 11 | 31 | 2 | 1 | 24+3 | 37+3 | yes |
| 12 | 28 | 1 | 0 | 24+3 | 38+3 | yes |
| 13 | 29 | 1 | 0 | 24+4 | 37+3 | yes |
| 14 | 26 | 1 | 0 | 24+5 | 39+6 | yes |
| 15 | 28 | 1 | 0 | 24+5 | 39 | yes |
| 16 | 29 | 1 | 0 | 24+6 | 39+6 | yes |
| 17 | 34 | 3 | 1 | 25+1 | 38+5 | yes |
| 18 | 31 | 1 | 0 | 25+2 | 39+1 | yes |
| 19 | 31 | 3 | 1 | 25+3 | 40+4 | yes |
| 20 | 31 | 1 | 0 | 26+3 | 40 | yes |

## **1.2 Comparison of maternal age and sampling gestational** **week between cervical cerclage group and control group**

| group | N | Age(year),  Median(p25,p75) | *p* | Sampling gestational week,  Median(p25,p75) | *p* |
| --- | --- | --- | --- | --- | --- |
| Control group | 20 | 30.0(28.0,31.0) | 0.101 | 24.3(22.5,24.8) | 0.789 |
| Cervical cerclage | 30 | 31.5(28.7,33.2) |  | 24.2(22.6,25.0) |  |

**Supplementary table2 The sequence summary of all samples**

| **Sample**  **ID** | **Nochime/Total_Reads(%)** | **Total_**  **reads** | **Q20**  **(%)** | **Q30**  **(%)** | **Combined_reads** | **Qualified_reads** | **Nochime**  **_reads** | **Nochime**  **_Base(bp)** | **Nochime_AvgLen(bp)** | **Nochime_GC(%)** |
| --- | --- | --- | --- | --- | --- | --- | --- | --- | --- | --- |
| C1-02 | 74.98 | 154366 | 95.37 | 89.89 | 121825 | 119698 | 115751 | 49076049 | 423 | 51.62 |
| C1-03 | 71.24 | 178073 | 93.94 | 87.61 | 129416 | 126906 | 126863 | 53795138 | 424 | 51.19 |
| C1-04 | 71.98 | 175431 | 94.27 | 88.10 | 129631 | 127365 | 126280 | 53599153 | 424 | 51.27 |
| C1-05 | 67.88 | 278681 | 94.38 | 88.17 | 197266 | 193402 | 189155 | 76863471 | 406 | 57.40 |
| C1-06 | 68.57 | 181106 | 93.72 | 87.25 | 127141 | 124230 | 124184 | 52505466 | 422 | 51.23 |
| C1-07 | 69.83 | 541219 | 95.64 | 90.16 | 409842 | 399944 | 377952 | 154841389 | 409 | 56.37 |
| C1-08 | 71.53 | 126837 | 94.36 | 88.03 | 94239 | 93025 | 90721 | 37811683 | 416 | 53.77 |
| C1-09 | 76.90 | 164579 | 96.25 | 91.16 | 137430 | 135491 | 126557 | 53745474 | 424 | 50.89 |
| C1-10 | 73.03 | 211843 | 94.58 | 88.41 | 161124 | 159202 | 154707 | 64714362 | 418 | 53.23 |
| C1-11 | 64.69 | 194508 | 94.11 | 87.66 | 138887 | 136502 | 125836 | 53495547 | 425 | 52.29 |
| C1-12 | 81.49 | 191575 | 96.30 | 91.35 | 158270 | 156328 | 156114 | 66371704 | 425 | 51.58 |
| C1-13 | 68.26 | 161572 | 94.25 | 87.86 | 115068 | 113268 | 110290 | 46474984 | 421 | 52.54 |
| C1-14 | 69.11 | 224456 | 94.65 | 88.66 | 168113 | 165851 | 155131 | 65943437 | 425 | 51.10 |
| C1-15 | 68.14 | 123498 | 94.16 | 87.93 | 94810 | 92966 | 84157 | 35688149 | 424 | 51.31 |
| C1-16 | 73.21 | 179614 | 94.21 | 87.93 | 134196 | 131568 | 131489 | 55808095 | 424 | 51.67 |
| C1-17 | 81.01 | 117339 | 95.75 | 90.21 | 97782 | 96259 | 95052 | 40302421 | 424 | 51.46 |
| C1-18 | 82.91 | 178086 | 95.96 | 90.65 | 149764 | 147760 | 147646 | 62705622 | 424 | 51.66 |
| C1-19 | 81.99 | 146730 | 95.91 | 90.71 | 123406 | 121475 | 120309 | 51074639 | 424 | 51.26 |
| C1-20 | 63.94 | 596130 | 95.63 | 90.39 | 417520 | 395483 | 381194 | 159110202 | 417 | 51.61 |
| C1-21 | 74.73 | 216894 | 94.34 | 88.14 | 164539 | 162163 | 162088 | 68939561 | 425 | 51.20 |
| C1-22 | 80.09 | 216760 | 96.17 | 91.09 | 176494 | 174279 | 173596 | 73791396 | 425 | 51.25 |
| C1-23 | 56.33 | 274523 | 93.36 | 86.46 | 161504 | 157346 | 154639 | 64493028 | 417 | 52.73 |
| C1-24 | 80.55 | 90797 | 95.35 | 89.62 | 74633 | 73234 | 73137 | 31033135 | 424 | 51.73 |
| C1-25 | 65.93 | 147254 | 94.26 | 88.01 | 98878 | 97142 | 97091 | 41219180 | 424 | 51.65 |
| C1-26 | 80.41 | 39867 | 95.66 | 90.05 | 32609 | 32101 | 32056 | 13634838 | 425 | 51.20 |
| C1-27 | 68.26 | 165274 | 94.59 | 88.66 | 125975 | 124162 | 112811 | 47940615 | 424 | 51.47 |
| C1-28 | 72.35 | 225061 | 95.44 | 90.06 | 165628 | 162923 | 162835 | 69240627 | 425 | 52.37 |
| C1-29 | 82.95 | 103188 | 96.03 | 90.80 | 86883 | 85662 | 85593 | 36391248 | 425 | 51.59 |
| C1-30 | 71.98 | 296990 | 94.50 | 88.38 | 216921 | 213863 | 213775 | 90975896 | 425 | 51.59 |
| C1-31 | 79.78 | 148076 | 95.77 | 90.57 | 120372 | 118652 | 118141 | 48488609 | 410 | 56.51 |
| C2-02 | 25.04 | 264313 | 91.61 | 84.66 | 86849 | 66470 | 66174 | 23757556 | 359 | 52.65 |
| C2-03 | 14.99 | 211496 | 91.58 | 84.59 | 40036 | 31728 | 31709 | 11205804 | 353 | 52.07 |
| C2-05 | 63.80 | 418352 | 94.07 | 87.21 | 281195 | 272804 | 266898 | 108364998 | 406 | 57.45 |
| C2-06 | 44.58 | 198541 | 93.40 | 86.87 | 92161 | 88758 | 88506 | 37042808 | 418 | 48.41 |
| C2-08 | 71.92 | 189784 | 94.46 | 88.05 | 144965 | 142895 | 136500 | 56695064 | 415 | 54.13 |
| C2-09 | 48.85 | 215868 | 94.67 | 89.27 | 111404 | 106669 | 105442 | 42154756 | 399 | 49.21 |
| C2-10 | 57.32 | 269016 | 93.78 | 87.50 | 160237 | 154848 | 154191 | 65200756 | 422 | 52.58 |
| C2-11 | 68.40 | 168485 | 93.72 | 87.18 | 120722 | 118655 | 115245 | 48969493 | 424 | 51.44 |
| C2-12 | 26.61 | 195215 | 91.38 | 84.85 | 57914 | 52054 | 51941 | 20498198 | 394 | 54.13 |
| C2-13 | 59.65 | 211900 | 93.64 | 87.13 | 132070 | 128636 | 126402 | 53472180 | 423 | 54.27 |
| C2-14 | 70.55 | 214955 | 94.29 | 88.14 | 154998 | 152040 | 151650 | 64322292 | 424 | 51.24 |
| C2-15 | 65.62 | 296297 | 94.14 | 88.00 | 202775 | 198484 | 194417 | 82316270 | 423 | 53.36 |
| C2-16 | 47.60 | 69248 | 93.92 | 88.42 | 36147 | 33829 | 32959 | 13536705 | 410 | 52.85 |
| C2-17 | 68.46 | 388703 | 93.82 | 86.78 | 281456 | 277485 | 266104 | 110508233 | 415 | 53.55 |
| C2-18 | 66.80 | 191143 | 95.30 | 90.13 | 133277 | 131014 | 127689 | 54088346 | 423 | 53.11 |
| C2-19 | 78.68 | 157754 | 95.83 | 90.58 | 126848 | 124597 | 124119 | 52035926 | 419 | 50.66 |
| C2-20 | 37.84 | 143826 | 93.48 | 87.95 | 61993 | 55711 | 54426 | 21938924 | 403 | 52.25 |
| C2-21 | 67.88 | 313942 | 94.69 | 88.49 | 230402 | 216628 | 213109 | 88469655 | 415 | 54.92 |
| C2-22 | 83.28 | 195941 | 96.11 | 90.95 | 166295 | 163974 | 163188 | 69234928 | 424 | 50.52 |
| C2-23 | 13.08 | 251112 | 89.78 | 82.68 | 37578 | 32866 | 32846 | 12639345 | 384 | 52.67 |
| C2-24 | 80.03 | 163905 | 95.90 | 90.62 | 133150 | 131212 | 131168 | 55727919 | 424 | 51.73 |
| C2-25 | 47.70 | 214783 | 93.70 | 87.45 | 105237 | 102698 | 102441 | 43219318 | 421 | 53.58 |
| C2-26 | 46.25 | 99244 | 94.30 | 88.84 | 47318 | 45985 | 45897 | 19365982 | 421 | 52.10 |
| C2-27 | 12.24 | 269995 | 90.43 | 83.14 | 40409 | 33055 | 33040 | 12005985 | 363 | 52.26 |
| C2-28 | 61.69 | 297829 | 94.42 | 88.89 | 187388 | 184194 | 183737 | 77816303 | 423 | 51.33 |
| C2-29 | 80.83 | 116060 | 96.01 | 90.56 | 99723 | 97658 | 93813 | 39639001 | 422 | 52.42 |
| C2-30 | 27.92 | 228215 | 93.53 | 87.77 | 72298 | 64773 | 63721 | 25645033 | 402 | 52.75 |
| C2-31 | 76.91 | 106023 | 95.50 | 90.36 | 83972 | 82634 | 81539 | 33565909 | 411 | 56.42 |
| N-01 | 65.50 | 287107 | 95.94 | 91.26 | 198260 | 191619 | 188053 | 76789904 | 408 | 57.75 |
| N-02 | 69.84 | 154125 | 95.84 | 90.74 | 116468 | 114236 | 107646 | 45570218 | 423 | 51.49 |
| N-03 | 78.69 | 189807 | 95.77 | 90.52 | 152658 | 149789 | 149353 | 63289777 | 423 | 51.24 |
| N-04 | 75.74 | 301090 | 96.37 | 91.51 | 241990 | 238507 | 228046 | 96764085 | 424 | 51.42 |
| N-05 | 75.36 | 216145 | 95.94 | 90.77 | 165992 | 163089 | 162882 | 69047156 | 423 | 50.98 |
| N-06 | 69.92 | 166255 | 95.58 | 90.64 | 119451 | 117653 | 116239 | 49300986 | 424 | 51.80 |
| N-07 | 82.82 | 177656 | 96.45 | 91.37 | 151370 | 149826 | 147126 | 61712917 | 419 | 52.70 |
| N-08 | 70.44 | 214432 | 94.15 | 87.69 | 154298 | 151456 | 151051 | 64160698 | 424 | 51.22 |
| N-09 | 75.32 | 121392 | 96.68 | 92.79 | 94872 | 92367 | 91436 | 37483847 | 409 | 57.23 |
| N-10 | 87.70 | 160326 | 97.20 | 93.17 | 144893 | 142225 | 140612 | 59079088 | 420 | 52.56 |
| N-11 | 70.29 | 177282 | 95.03 | 89.68 | 129035 | 124998 | 124614 | 50467725 | 404 | 59.13 |
| N-12 | 70.50 | 210366 | 95.57 | 90.51 | 163333 | 160685 | 148317 | 62829339 | 423 | 51.58 |
| N-13 | 78.99 | 188242 | 96.33 | 91.44 | 151378 | 149587 | 148689 | 63202335 | 425 | 51.69 |
| N-14 | 67.30 | 241295 | 95.68 | 90.45 | 175511 | 171717 | 162397 | 68588174 | 422 | 51.01 |
| N-15 | 71.88 | 148148 | 95.57 | 90.12 | 109162 | 106676 | 106488 | 45083612 | 423 | 50.90 |
| N-16 | 75.22 | 169344 | 95.97 | 90.96 | 130305 | 128438 | 127376 | 54088514 | 424 | 51.72 |
| N-17 | 79.91 | 173413 | 96.42 | 91.29 | 144493 | 142692 | 138570 | 57936315 | 418 | 53.11 |
| N-18 | 88.83 | 144176 | 97.28 | 93.49 | 130368 | 128229 | 128069 | 54506148 | 425 | 51.20 |
| N-19 | 87.02 | 181688 | 97.23 | 93.63 | 161628 | 158885 | 158103 | 64179805 | 405 | 57.86 |
| N-20 | 86.95 | 168096 | 97.04 | 93.05 | 149995 | 147238 | 146152 | 60799124 | 415 | 54.08 |

## **Supplementary table 3**

## **Comparison of α diversity between groups**

|  | Chao1 | | | Shannon | | | Inverse Simpson | | |
| --- | --- | --- | --- | --- | --- | --- | --- | --- | --- |
|  | Chao1 | | Kruskal  /wilcox | Shannon | | Kruskal  /wilcox | Inverse Simpson | | Kruskal  /wilcox |
| Control *VS* Cerclage1 | control | Cerclage1 | *p value* | control | Cerclage1 | *p value* | control | Cerclage1 | *p value* |
|  | 248.36 | 260.06 | *Wilcoxon 0.161* | 0.84 | 0.69 | *Wilcoxon0.173* | 0.29 | 0.24 | *Wilcoxon*  *0.183* |
| Cerclage1  term *VS* preterm | term | preterm | *p value* | term | preterm | *p value* | term | preterm | *p value* |
|  | 296.45 | 227.73 | *Wilcoxon 0.172* | 0.47 | 0.84 | *Wilcoxon0.051* | 0.14 | 0.31 | *Wilcoxon*  *0.031* |
| Nonemergency  Cerclage1 *VS* Cerclage2 | Cerclage1 | Cerclage2 | *p value* | Cerclage1 | Cerclage2 | *p value* | Cerclage1 | Cerclage2 | *p value* |
|  | 248.0667 | 387.3828 | *Wilcoxon 1.000* | 0.7912534 | 1.3621818 | *Wilcoxon0.332* | 0.2883674 | 0.4028743 | *Wilcoxon*  *0.484* |
| Emergency  Cerclage1 *VS* Cerclage2 | Cerclage1 | Cerclage2 | *p value* | Cerclage1 | Cerclage2 | *p value* | Cerclage1 | Cerclage2 | *p value* |
|  | 267.011 | 291.4541 | *Wilcoxon0.933* | 0.6344117 | 1.5471147 | *Wilcoxon0.004* | 0.2144076 | 0.4299875 | *Wilcoxon*  *0.015* |

## **Comparison of β diversity between groups**

|  | anosim | mrpp | adonis | amova |
| --- | --- | --- | --- | --- |
| Control vs Cerclage1 | *R=0.033*  *p=0.192* | *A=0.284*  *p=0.295* | *R^2^=0.029(0.971)*  *p=0.244* | *Fs=1.445*  *p=0.219* |
| Cerclage1  term vs preterm | *R=-0.0412*  *p=0.797* | *A=0.25*  *p=0.408* | *R^2^=0.026(0.973)*  *p=0.412* | *Fs=0.793*  *p=0.403* |
| Nonemergency  Cerclage1 *VS* Cerclage2 | *R=0.027*  *p=0.238* | *A=0.483*  *p=0.211* | *R^2^=0.057(0.942)*  *p=0.262* | *Fs=1.222*  *p=0.274* |
| Emergency  Cerclage1 *VS* Cerclage2 | *R=0.210*  *p=0.001* | *A=0.297*  *p=0.003* | *R^2^=0.117(0.883)*  *p=0.008* | *Fs=4.511*  *p=0.002* |
